# Identification of drought-responsive miRNAs and physiological characterization of tea plant (*Camellia sinensis* L.) under drought stress

**DOI:** 10.1186/s12870-017-1172-6

**Published:** 2017-11-21

**Authors:** Yuqiong Guo, Shanshan Zhao, Chen Zhu, Xiaojun Chang, Chuan Yue, Zhong Wang, Yuling Lin, Zhongxiong Lai

**Affiliations:** 1 College of Horticulture, Fujian Agriculture and Forestry University, Fuzhou, 350002 China; 2 Institute of Horticultural Biotechnology, Fujian Agriculture and Forestry University, Fuzhou, 350002 China

**Keywords:** *Camellia sinensis*, Drought stress, miRNA, Physiological characteristics

## Abstract

**Background:**

Drought stress is one of the major natural challenges in the main tea-producing regions of China. The tea plant (*Camellia sinensis*) is a traditional beverage plant whose growth status directly affects tea quality. Recent studies have revealed that microRNAs (miRNAs) play key functions in plant growth and development. Although some miRNAs have been identified in *C. sinensis*, little is known about their roles in the drought stress response of tea plants.

**Results:**

Physiological characterization of *Camellia sinensis* ‘Tieguanyin’ under drought stress showed that the malondialdehyde concentration and electrical conductivity of leaves of drought-stressed plants increased when the chlorophyll concentration decreased under severe drought stress. We sequenced four small-RNA (sRNA) libraries constructed from leaves of plants subjected to four different treatments, normal water supply (CK); mild drought stress (T1); moderate drought stress (T2) and severe drought stress (T3). A total of 299 known mature miRNA sequences and 46 novel miRNAs were identified. Gene Ontology enrichment analysis revealed that most of the differentially expressed-miRNA target genes were related to regulation of transcription. Kyoto Encyclopedia of Genes and Genomes analysis revealed that the most highly enriched pathways under drought stress were D-alanine metabolism, sulfur metabolism, and mineral absorption pathways. Real-time quantitative PCR (qPCR) was used to validate the expression patterns of 21 miRNAs (2 up-regulated and 19 down-regulated under drought stress). The observed co-regulation of the miR166 family and their targets *ATHB-14-like* and *ATHB-15-like* indicate the presence of negative feedback regulation in miRNA pathways.

**Conclusions:**

Analyses of drought-responsive miRNAs in tea plants showed that most of differentially expressed-miRNA target genes were related to regulation of transcription. The results of study revealed that the expressions of phase-specific miRNAs vary with morphological, physiological, and biochemical changes. These findings will be useful for research on drought resistance and provide insights into the mechanisms of drought adaptation and resistance in *C. sinensis*.

**Electronic supplementary material:**

The online version of this article (10.1186/s12870-017-1172-6) contains supplementary material, which is available to authorized users.

## Background

Tea (*Camellia sinensis* L.) is an economically important crop from southwestern China whose leaves are the source of one of the most popular non-alcoholic beverages worldwide. Tea leaf quality and yield mainly depend on the growth status of tea plants. In the main tea-producing regions of China, which are spread over the middle and lower reaches of the Yangtze River, tea trees are under drought stress during the tea production period, especially in summer and fall. Drought stress has been reported to reduce tea production by 14%–33% and to increase tea plant mortality by 6%–19% [1]. It is urgent, therefore, to determine how tea plants respond to drought stress.

Increasing attention is being paid to the drought tolerance of tea plants. Several studies have found that tea plants adapt to drought stress by various morphological changes, osmotic regulation, reactive oxygen species scavenging, and plant hormone regulation [2–4]. In addition, amplified fragment length polymorphism (AFLP) and suppression subtractive hybridization (SSH) analyses have been used to identify genes related to the drought response of *C. sinensis* [5–7]. Transcriptome analyses have been used to investigate gene regulation under drought conditions, resulting in the identification of many genes involved in the drought response [8, 9]. Despite these advances, there is still much to learn about the regulation of the plant drought response.

Apart from the series of physiological changes mentioned above, miRNAs have been shown to be involved in drought stress responses in some plants. However, the miRNA-associated regulatory networks in *C. sinensis* remain to be uncovered. Hence, the response of miRNAs in *C. sinensis* to stress and their roles in adaptation and tolerance have become major research topics [10–12]. Plant miRNAs are known have important functions in responses to biotic and abiotic stresses [13–24]. Studies have shown that miRNAs function as important modulators of drought tolerance by influencing the cleavage of drought-responsive genes or inhibiting their translation. Many of these miRNAs target genes encoding transcription factors; therefore, miRNAs function at the center of drought-stress regulatory networks [25–27]. Thus, the identification of miRNAs and their target genes is essential to reveal the molecular mechanism of miRNAs in the drought stress response.

Numerous conserved miRNAs and their targets have recently been identified in *C. sinensis* [28–30]. Some of these miRNAs are responsive to dormancy [29] and cold stress [30]. Using small RNA (sRNA) sequencing, Liu and Xu et al. [31] identified 268 conserved and 62 novel miRNAs from the drought-resistant tea cultivar ‘Ningzhou2’ and the drought-susceptible tea cultivar ‘Zhuyeqi’. While there is a large amount of information on miRNA expression under drought stress in other woody plants, relatively little information is available for tea plants. In the present study, we used Illumina HiSeq 2500 technology to identify putative miRNAs and investigated their expression profiles in the unique oolong tea cultivar ‘Tieguanyin’ under four drought stress conditions. We predicted the target genes of the miRNAs related to the drought stress response in *C. sinensis*. We also analyzed physiological characteristics of drought-stressed and control plants, including malondialdehyde (MDA) concentration, electrical conductivity, chlorophyll concentration, and leaf water content. Our results provide new information about the regulatory mechanism(s) of miRNAs in the drought stress response of tea plants.

## Methods

### Plant materials and drought stress treatments

**Fig. 1:**
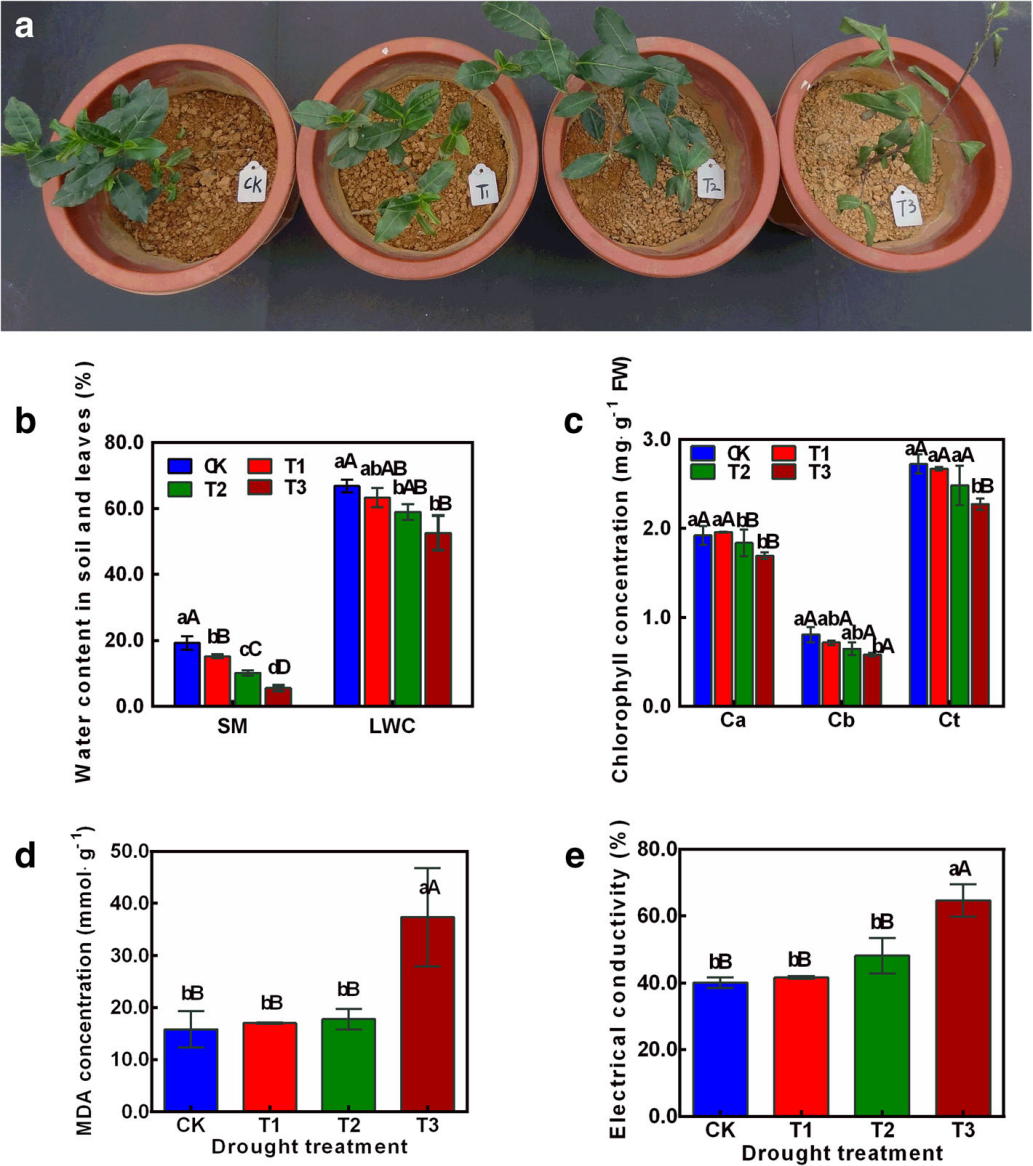
Physiological changes in *Camellia sinensis* ‘Tieguanyin’ plants subjected to different drought stress conditions. **a** Phenotypes of tea plants after exposure to drought stress for 10 days. CK: normal water supply; T1: mild drought stress; T2: moderate drought stress; T3: severe drought stress. **b**–**e** Soil moisture (SM) and leaf water content (LWC) (**b**), chlorophyll a (Ca), chlorophyll b (Cb), and total chlorophyll (Ct) concentration (**c**), malondialdehyde (MDA) concentration (**d**), and electrical conductivity (**e**) of tea plants at different drought stress stages. Data are means ± SE (*n* = 3)

The experimental material was *C. sinensis* ‘Tieguanyin’, the most well-known oolong tea cultivar. Healthy 1-year-old tea plant cuttings were transplanted into pots and grown in a greenhouse under a 12-h light (30 °C)/12-h dark (20 °C) photoperiod and 40%–65% relative humidity. Drought treatments were applied using the compensatory watering method, with all other ambient conditions held constant. There were four levels of drought treatment (Fig. 1a): normal water supply (soil moisture content = 19.50%; CK), mild drought stress (soil moisture content = 15.20%; T1), moderate drought stress (soil moisture content = 10.17%; T2) and severe drought stress (soil moisture content = 5.54%; T3). Each treatment lasted for 10 days. In addition, three independent biological replicates were established for each drought treatment. Each replicate was collected from 10 randomly selected tea plants. The tender leaves of *C. sinensis* were sampled, frozen immediately in liquid nitrogen, and stored at −80 °C until further analysis.

### Determination of relative water content, electrical conductivity, and chlorophyll and MDA concentrations

Leaves were randomly sampled from plants in the drought treatments and their physiological characteristics were measured. To detect the effects of drought stress on tea plants, relative water contents (including soil moisture and leaf water content) were determined according to Upadhyaya et al. [32]. The leaf chlorophyll concentration was determined as previously described [33]; MDA was extracted and analyzed as described elsewhere [34], and leaf conductivity was measured as described by Luo et al. [35].

### Total RNA extraction

For miRNA sequencing, total RNA was extracted from tea leaves using a Trizol Reagent kit (Invitrogen/Life Technologies, Carlsbad, CA, USA) according to the manufacturer’s instructions. Total RNA quality was checked by 1% agarose gel electrophoresis and ultra micro-ultraviolet spectrophotometry. The RNA samples were stored at −80 °C until miRNA sequencing.

### Library preparation and sequencing

The four small RNA libraries generated respectively from four treated samples (CK, T1, T2 and T3) and sequenced by the Biomarker Technology Co. (Beijing, China, http://www.biomarker.com.cn/) using an Illumina HiSeq 2500 instrument. Total RNA was extracted from samples from each treatment, and linkers were added to the 5′ and 3′ ends of RNA by T4 RNA ligase. Then, the target fragments were amplified by reverse transcription PCR using synthesized first-strand cDNA as the template, and screened by polyacrylamide gel electrophoresis. Each small RNA library was constructed using the fragments reclaimed from the gel. Finally, each small RNA library was sequenced.

The candidate miRNA genes selected for further investigation were those that satisfied the following criteria, based on Meyers et al. [36]: (1) a mature sequence localized in one arm of the stem-loop structure and between 19 and 24 nt in length; (2) corresponding miRNA* sequence identified; (3) pre-miRNA sequence folded into an appropriate stem-loop hairpin secondary structure; (4) minimum free energy (MFE) of secondary structures ≤ −20 kcal/mol; and (5) no more than 4 nt mismatches in the miRNA:miRNA* duplex.

### MiRNA annotation and target gene prediction

To identify sRNAs originating from miRNA degradation fragments, exon and intron sense and antisense sequences were compared between sRNA and miRNAs using Overlap software. The identified sRNA sequences were successfully annotated using rRNA, scRNA, snoRNA, snRNA, and tRNA sequences from Rfam3 and GenBank databases. To ensure unique sRNA annotations, sequence comparisons were prioritized in the following order: rRNAs > conserved miRNAs > repeats > exons > introns.

The potential target genes of novel *C. sinensis* miRNAs were predicted using the algorithms of Allen [37] and Schwab [38], with the following criteria: (1) less than four mismatches between the sRNA and its target (mismatches in G-U bases were counted as 0.5); (2) less than two adjacent mismatches in the miRNA/target duplex; (3) less than 2.5 mismatches in positions 1–12 of the miRNA/target duplex (5′ end of the miRNA); (4) no adjacent mismatches in positions 2–12 of the miRNA/target duplex (5′ end of the miRNA); (5) no mismatches in positions 10–11 of the miRNA/target duplex; and (6) minimum free energy of the miRNA/target duplex ≥75% of the minimum free energy of the miRNA bound to its optimal complement.

### Real-time quantitative PCR (qPCR) detection of miRNAs and their targets in *C. sinensis*

The expression levels of relevant miRNAs and their target genes were analyzed by qPCR. The cDNA of miRNAs was synthesized using a SYBR One Step PrimeScript miRNA cDNA Synthesis kit (Takara, Otsu, Japan, Code: RR716). The qPCR analyses were conducted using SYBR Premix Ex *Taq* (Takara) on a LightCycler480 qPCR instrument (Roche Applied Sciences, Basel, Switzerland) under the following cycling conditions: preheating at 94 °C for 10 s, followed by 50 cycles of 94 °C for 5 s, 57–62 °C for 15 s and 72 °C for 10 s, with a final step at 40 °C for 30 s. A melting curve analysis was performed to check for primer dimers. The primers used for qPCR are listed in Additional file 1: Table S1. All reactions were repeated in triplicate, with the 18S rRNA gene used as an internal control. The relative expression levels of miRNAs were calculated using the 2^−ΔΔCt^ method of Schmittgen et al. [39].

### Statistical analysis

Statistical analyses were conducted using SPSS version 13 (SPSS, Inc., Chicago, IL, USA). The data were analyzed by one-way analysis of variance followed by Tukey’s post-hoc test, with differences considered significant at *p* < 0.05.

## Results

### Changes in chlorophyll and MDA concentrations, and electrical conductivity under drought stress in *C. sinensis*

The phenotypes of *C. sinensis* leaves were recorded under conditions ranging from normal water supply to severe drought (CK, T1, T2, and T3). The leaves were slightly curled and deformed under T1, and dull, curled, and wilted under T2. With increasing severity of drought stress (T3), leaves wilted further and most branches died (Fig. 1a). The leaf water content (LWC), soil moisture (SM), chlorophyll and MDA concentrations, and electrical conductivity were measured under the four different treatments. The LWC may reflect metabolic intensity and, to some extent, indicate the water absorption capacity and dehydration tolerance under drought conditions. Compared with the LWC in CK, that in T1, T2, and T3 was decreased by 6.93%, 11.18%, and 18.86%, respectively. Chlorophyll a, chlorophyll b, and total chlorophyll concentrations decreased slightly, but differences among treatments were not significant (Fig. 1c). The MDA concentration and leaf electrical conductivity were markedly higher under the T3 treatment than under the other treatments (Fig. 1d–e).

### Construction and high-throughput sequencing of sRNA libraries from *C. sinensis*

To identify drought-responsive miRNAs in tea plants, four sRNA libraries generated from plants in the CK, T1, T2, and T3 treatments were sequenced using the Illumina platform. Table 1 provides a statistical summary of sequencing results for the four sRNA libraries.

**Table 1:** Summary of sequencing results of small RNA libraries constructed from Tieguanyin tea plants subjected to normal water supply (CK), mild drought stress (T1), moderate drought stress (T2) and severe drought stress (T3) treatments

Category	CK	T1	T2	T3
	Reads No.	Reads No.	Reads No.	Reads No.
	(Percentage)	(Percentage)	(Percentage)	(Percentage)
Total_reads	15,843,812	13,383,907	14,689,202	13,704,671
High_quality	15,799,925	13,273,561	14,569,424	13,594,243
	(100%)	(100%)	(100%)	(100%)
3’adapter_null	3287	10,422	11,340	8303
	(0.02%)	(0.08%)	(0.08%)	(0.06%)
Insert_null	1774	2598	2888	9478
	(0.01%)	(0.02%)	(0.02%)	(0.07%)
5’adapter_contaminants	10,395	28,504	31,895	78,829
	(0.07%)	(0.21%)	(0.22%)	(0.58%)
Smaller_than 18nt	3008	10,268	9309	42,893
	(0.02%)	(0.08%)	(0.06%)	(0.32%)
PolyA	3688	3372	3630	2612
	(0.02%)	(0.03%)	(0.02%)	(0.02%)
Clean_reads	15,777,773	13,218,397	14,510,362	13,452,128
	(99.86%)	(99.58%)	(99.59%)	(98.95%)

After removing 5′-adapters, 3′-adapters, and low-quality reads, we obtained 15,777,773, 13,218,397, 14,510,362, and 13,452,128 clean reads from the CK, T1, T2, and T3 samples, respectively. The proportion of clean reads out of total sequencing reads was 99.86%, 99.58%, 99.58%, and 98.95% for CK, T1, T2, and T3, respectively, indicating that the read quality was sufficient for further analyses.

**Fig. 2:**
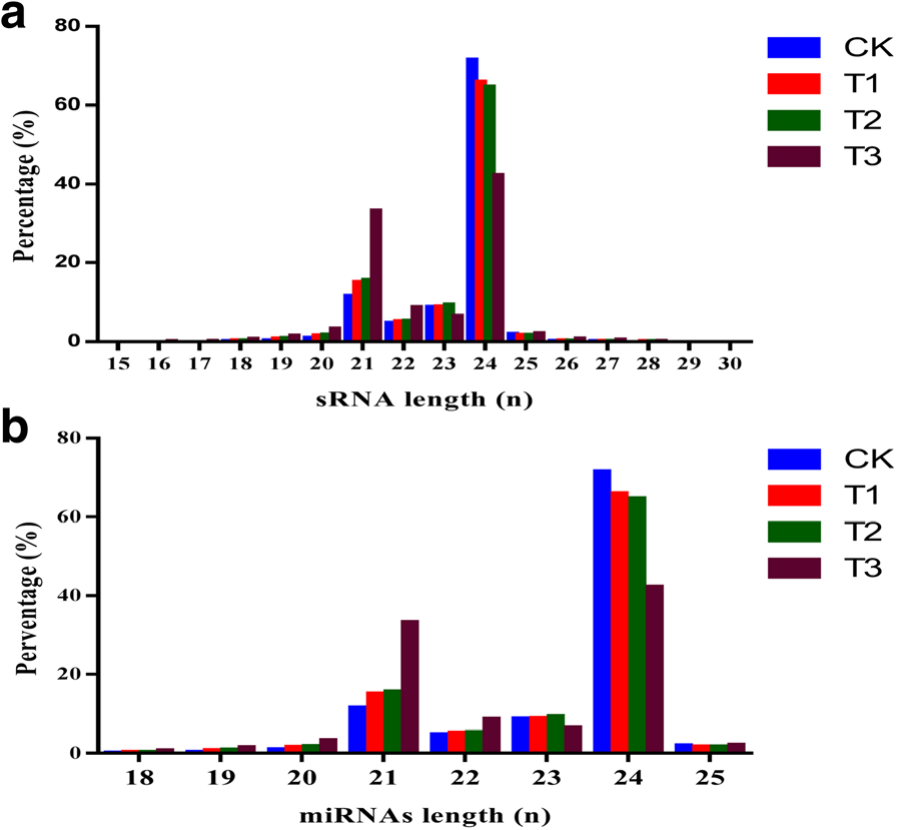
Size distributions of sRNAs (**a**) and miRNAs (**b**) in *Camellia sinensis* plants under different drought stress conditions

As shown in Fig. 2a, the lengths of all *C. sinensis* sRNA sequences ranged between 21 and 24 nt. As the severity of drought stress increased, the number of 21-nt sRNA sequences increased (by 3.97%, 4.52%, and 22% in T1, T2, and T3, respectively, compared with CK) while the number of 24-nt sRNAs decreased.

### Classification and annotation of sRNAs under drought stress in *C. sinensis*

Comparison with precursor miRNAs and mature miRNAs of all plants in miRBase 21 (http://www.mirbase.org/) revealed that 32,364 (0.37%), 29,770 (0.41%), 35,077 (0.45%), and 30,580 (0.59%) unique sequences from CK, T1, T2, and T3, respectively, were similar to known miRNAs [40]. Other unique sequences, such as rRNAs (CK: 37,966, 0.43%; T1: 56,302, 0.77%; T2: 55,779, 0.71%; T3: 120,595, 2.33%), snRNAs (CK: 1969, 0.02%; T1: 2062, 0.03%; T2: 2363, 0.03%; T3: 3289, 0.06%), snoRNAs (CK: 685, 0.01%; T1: 745, 0.01%; T2: 883, 0.01%; T3: 892, 0.02%), and tRNAs (CK: 2632, 0.03%; T1: 4201, 0.06%; T2: 4291, 0.05%; T3: 8184, 0.16%), were also characterized according to the Rfam database (http://rfam.xfam.org/) [41]. The distribution of characterized sequences from the four libraries is shown in Table 2.

**Table 2:** Distribution of unique small RNA (sRNA) sequences from Tieguanyin tea plants subjected to normal water supply (CK), mild drought stress (T1), moderate drought stress (T2) and severe drought stress (T3) treatments. “Unann” refers to sRNA sequences for which no annotation information could be obtained by database comparisons

Types	CK	T1	T2	T3
	UniquesRNAs	Unique sRNAs	Unique sRNAs	Unique sRNAs
	(Percentage)	(Percentage)	(Percentage)	(Percentage)
Total	8,797,521	7,277,866	7,842,237	5,165,455
	(100%)	(100%)	(100%)	(100%)
Exon_antisense	743	842	897	1071
	(0.01%)	(0.01%)	(0.01%)	(0.02%)
Exon_sense	1086	1354	1443	2136
	(0.01%)	(0.02%)	(0.02%)	(0.04%)
Intron_antisense	156	180	164	227
	(0%)	(0%)	(0%)	(0%)
Intron_sense	189	211	205	287
	(0%)	(0%)	(0%)	(0%)
miRNA	32,364	29,770	35,077	30,580
	(0.37%)	(0.41%)	(0.45%)	(0.59%)
rRNA	37,966	56,302	55,779	120,595
	(0.43%)	(0.77%)	(0.71%)	(2.33%)
Repeat	31,260	28,218	29,298	26,544
	(0.36%)	(0.39%)	(0.37%)	(0.51%)
snRNA	1969	2062	2363	3289
	(0.02%)	(0.03%)	(0.03%)	(0.06%)
snoRNA	685	745	883	892
	(0.01%)	(0.01%)	(0.01%)	(0.02%)
tRNA	2632	4201	4291	8184
	(0.03%)	(0.06%)	(0.05%)	(0.16%)
unann	8,688,471	7,153,981	7,711,837	4,971,650
	(98.76%)	(98.30%)	(98.34%)	(96.25%)

### Identification of conserved miRNAs during drought stress in *C. sinensis*

A total of 191 known mature miRNAs were identified in *C. sinensis*: 117 from CK, 127 from T1, 127 from T2, and 129 from T3, with 103 common to all four libraries. The number of miRNA target genes predicted in CK, T1, T2, and T3 was 2078, 2077, 2778, and 2064, respectively (Table 3). In total, 4376 target genes of 191 conserved miRNAs were obtained from the four treatment samples.

**Table 3:** Number of predicted target genes of conserved and novel miRNAs and novel miRNA precursor candidates. Each entry in the row labeled “Novel miRNAs No.” refers to the total number of novel miRNAs followed by the number of novel miRNAs with predicted target gene loci

Samples	CK	T1	T2	T3	Total
Conserved miRNAs No.	117	127	127	129	191
Predicted target genes No. of conserved miRNAs	2078	2077	2778	2064	4376
Novel miRNAs No.	34/29	61/48	46/43	57/52	87/59
Predicted target genes No. of novel miRNAs	1535	1341	2597	757	5528

**Fig. 3:**
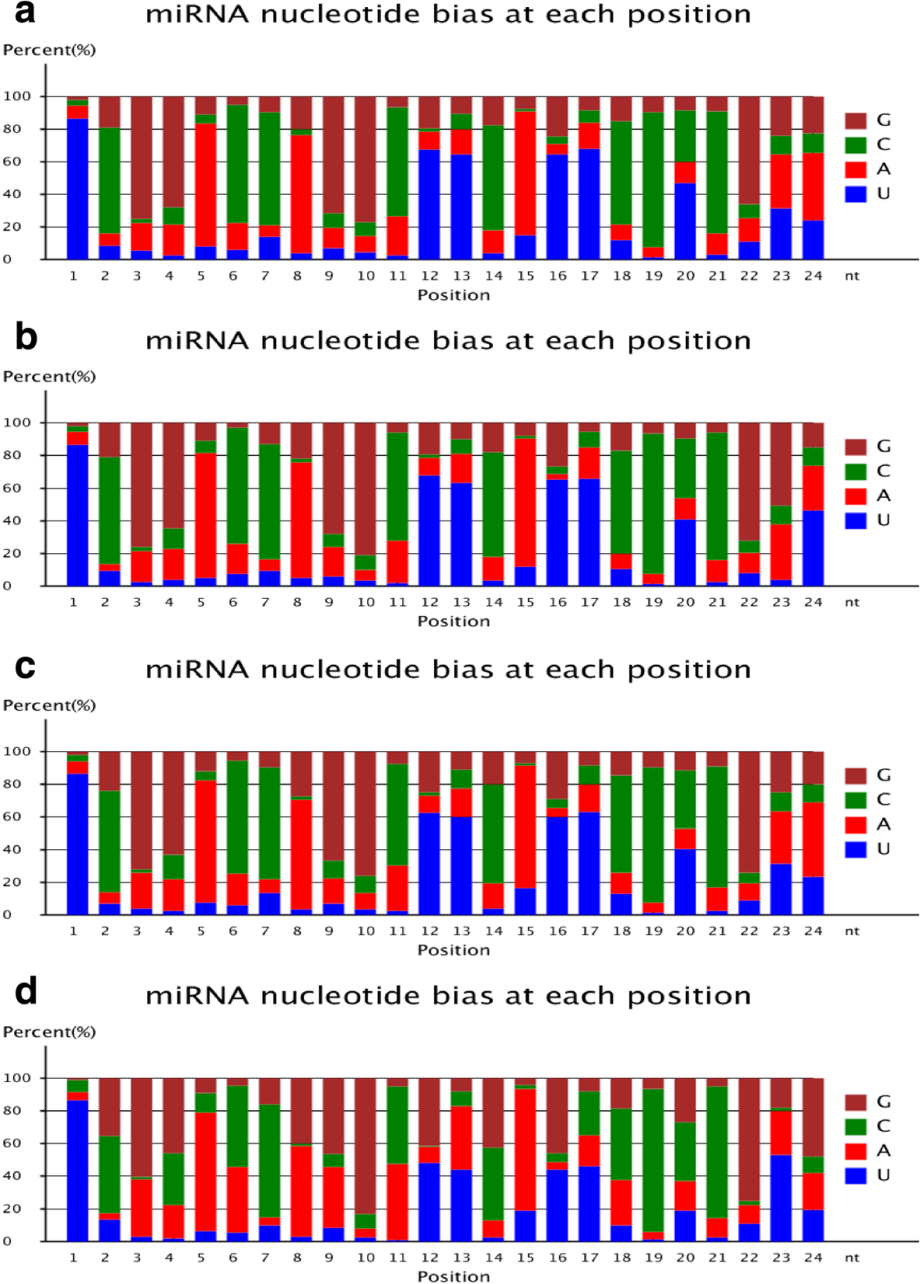
Nucleotide bias at each position of *Camellia sinensis* conserved miRNAs under different drought stress conditions. Drought stress conditions were as follows: **a** normal water supply, **b** mild drought stress, **c** moderate drought stress, and **d** severe drought stress. X-axis represents miRNA nucleotide positions; y-axis indicates percentage of each specific nucleotide at each nucleotide position. Brown, green, red, and blue correspond to guanine (G), cytosine (C), adenine (A), and uracil (U), respectively

Although the lengths of known *C. sinensis* miRNAs ranged from 18 to 25 nt, most were 24-nt (CK: 71.48%; T1: 65.87%; T2: 64.63%; T3: 42.26%) or 21-nt (CK: 11.50%; T1: 15.02%; T2: 15.57%; T3: 33.25%) (Fig. 2b). As shown in Fig. 3, the majority of miRNA sequences from the *C. sinensis* libraries started with uridine. The characteristics of *C. sinensis* miRNA sequences were consistent with those previously reported for miRNA sequences [42].

The percentages of adenine (A), uracil (U), guanine (G), and cytosine (C) at each locus of conserved miRNAs changed dynamically with increasing drought stress in Tieguanyin tea plants (Fig. 3). One of the most frequent bases in *C. sinensis* conserved miRNAs, G, was present mainly at nucleotide positions 3, 4, 9, 10, and 22, while U was found mainly at positions 1, 12, 13, 16, and 17. Another heavily represented base in conserved miRNAs was C, which was mainly distributed at positions 2, 6, 7, 11, 14, and 18–21.

### Prediction of novel miRNAs during drought stress in *C. sinensis*

To predict novel miRNAs, the *Arabidopsis thaliana* genome (ftp://ftp.arabidopsis.org/home/tair/Genes/TAIR10_genome_release) was selected as a reference. Comparisons of the sRNA data with those in the miRBase and other databases revealed 34, 61, 46, and 57 novel miRNAs from the CK, T1, T2, and T3 sample libraries, respectively. A total of 87 novel miRNAs were identified from the four Tieguanyin tea plant libraries, and 1535, 1341, 2597, and 757 putative target genes of 29, 48, 43, and 52 novel miRNAs were identified in the CK, T1, T2, and T3 libraries, respectively. Overall, 5528 target genes of 59 novel miRNAs were predicted (Table 3).

**Fig. 4:**
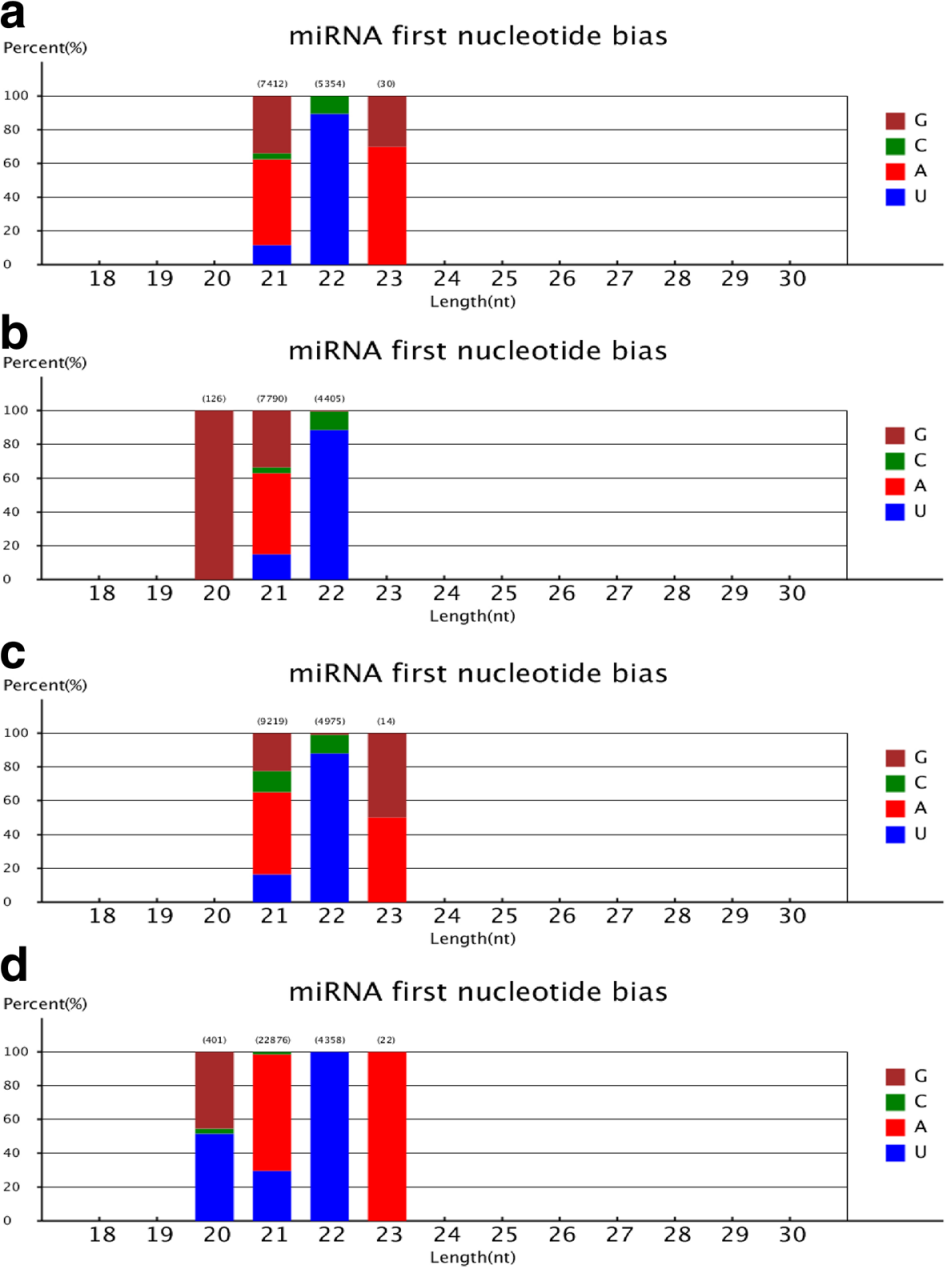
First nucleotide bias of length classes of *Camellia sinensis* novel miRNAs under drought stress conditions. Drought stress conditions were as follows: **a** normal water supply, CK; **b** mild drought stress, T1; **c** moderate drought stress, T2; and **d** severe drought stress, T3. Brown, green, red, and blue correspond to guanine (G), cytosine (C), adenosine (A), and uracil (U), respectively

The length range of novel miRNAs in the CK, T1, T2, and T3 libraries was 21–23, 20–22, 21–23, and 20–23 nt, respectively (Fig. 4). No novel 24-nt miRNAs were identified. Most novel miRNAs in *C. sinensis* were 21-nt: 7412 (57.92%) in CK, 7790 (63.23%) in T1, 9291 (65.06%) in T2, and 22,876 (82.71%) in T3.

To identify additional tea plant miRNAs, we used transcriptome data of *C. sinensis* Tieguanyin as a reference. Hierarchical indexing for spliced alignment of transcripts (Hisat) was used to calculate the coverage of the tea transcriptome data relative to the whole tea genomic data. Calculations were performed as described by Kim et al. [43]. The coverage percentage was 83.80%. According to the sequencing quality of the reference genome, generally more than 70% coverage is acceptable [43]. Using these analyses, we identified 176 miRNA sequencing reads and predicted 4067 target gene loci (Additional file 2: Table S2).

### Analysis of differentially expressed miRNAs during drought stress in *C. sinensis*

**Fig. 5:**
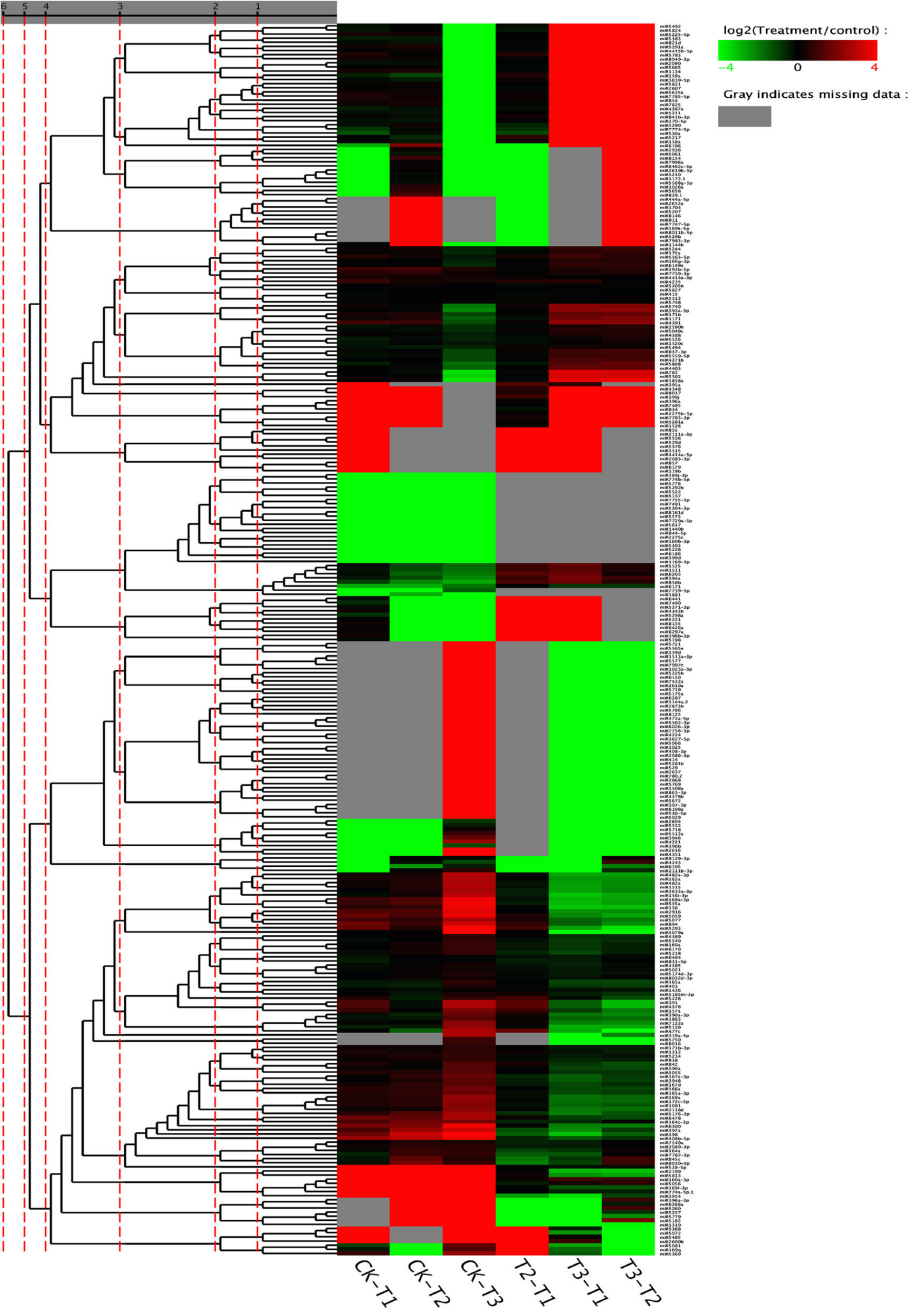
Heat map of *Camellia sinensis* conserved miRNAs differentially expressed between different drought stress conditions. Drought stress conditions were as follows: normal water supply (CK), mild drought stress (T1), moderate drought stress (T2), and severe drought stress (T3). Scale bar corresponds to miRNA relative expression levels; different hues indicate relative signal intensities. Red and green correspond respectively to up- or down-regulated expression of a given miRNA at each stage

The expression levels of *C. sinensis* miRNAs differed significantly among the drought treatments (Fig. 5; Additional file 3: Figure S1). In total, we identified 299 known mature miRNA sequences and 46 novel miRNAs. Of these known miRNAs, 101 were differentially expressed between CK and T1 (48 up-regulated and 53 down-regulated; Additional file 4: Table S3), and 108 between CK and T2 (55 up-regulated and 53 down-regulated; Additional file 5: Table S4). There were 211 differentially expressed miRNAs between CK and T3 (112 up-regulated and 99 down-regulated; Additional file 6: Table S5); 76 known miRNAs expressed only in CK, 61 expressed only in T3, 51 up-regulated in T3 compared with CK, and 23 down-regulated in T3 compared with CK.

### Gene ontology (GO) and Kyoto encyclopedia of genes and genomes (KEGG) pathway analyses of miRNAs expressed under drought stress in *C. sinensis*

**Fig. 6:**
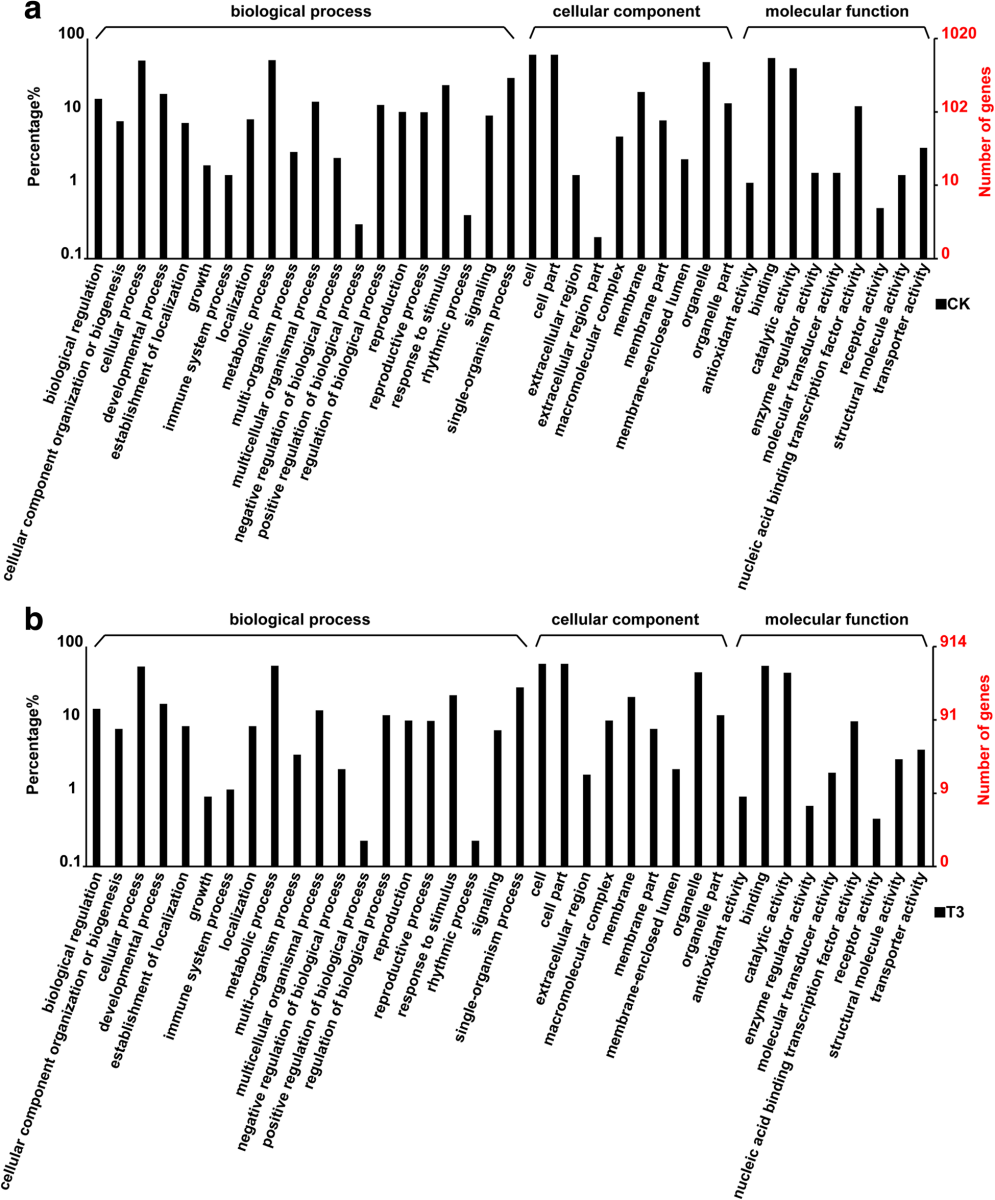
Gene Ontology annotations of predicted target genes identified from different drought stress treatments. **a** Normal water supply, CK; **b** severe drought stress, T3

Next, GO annotations were obtained from the Gene Ontology (http://www.geneontology.org/) and NCBI (ftp://ftp.ncbi.nih.gov/gene/DATA) databases. Gene annotations and classifications according to GO biological process, cellular component, and molecular function categories are provided in Fig. 6a, Additional file 7: Figure S2, Additional file 8: Figure S3, and Fig. 6b. The GO classifications statistics are provided in Additional file 9: Table S6. In these analyses, we assigned 6143, 6264, 6910, and 5389 unigenes in the CK, T1, T2, and T3 libraries, respectively. The top three subgroups in the biological process category were cellular process (1004 genes), metabolic process (968 genes), and single-organism process (607 genes). In the molecular function category, the top three subgroups were binding (1053 genes), catalytic activity (668 genes), and nucleic acid binding transcription factor activity (297 genes). The top three subgroups in the cellular component category were cell, cell part, and organelle, which accounted for 77.78%, 77.56%, 77.54%, and 75.13% of all unigenes in the CK, T1, T2, and T3 libraries, respectively.

**Fig. 7:**
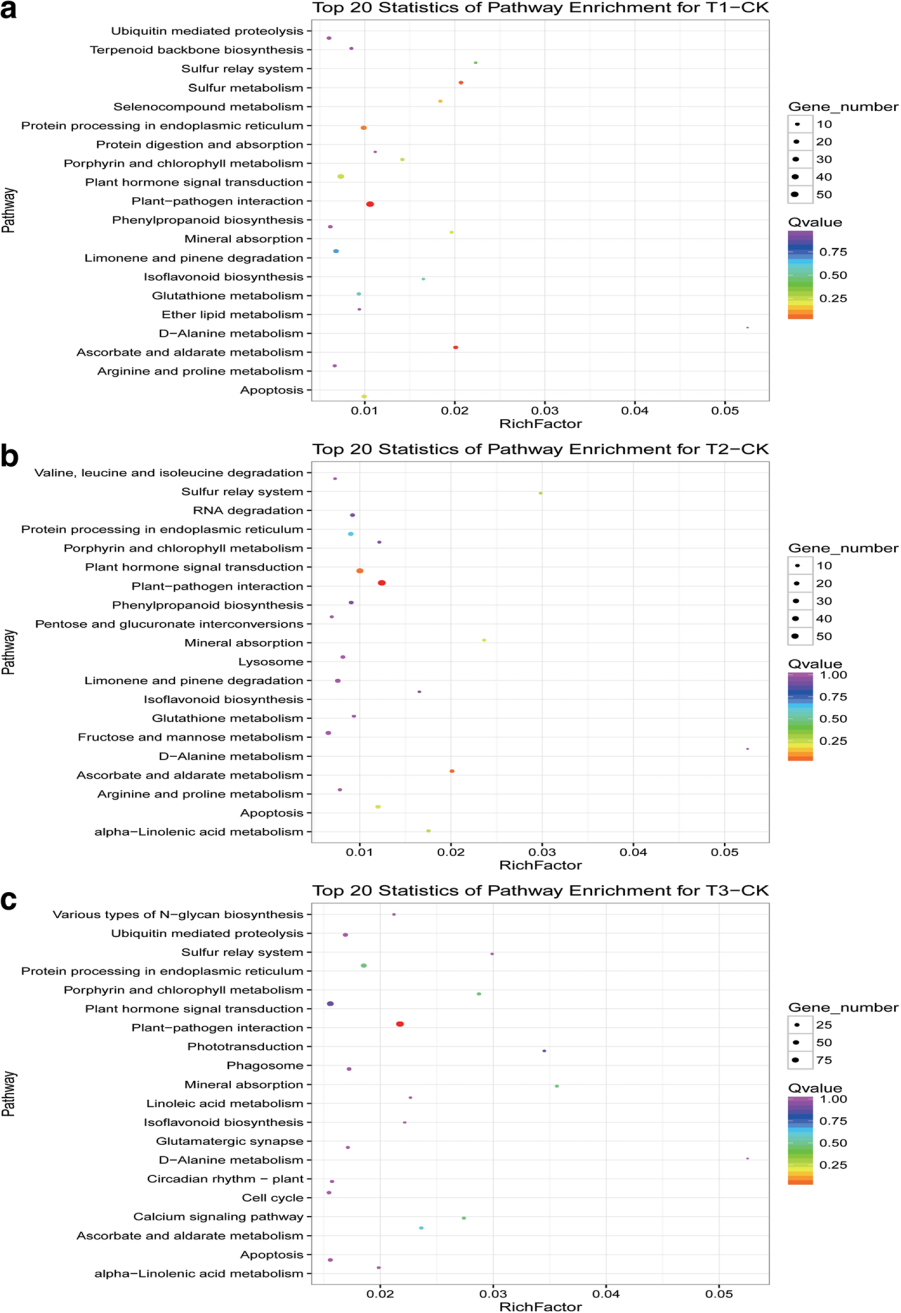
Top 20 KEGG pathways enriched in conserved miRNA target genes differentially expressed between treatments. **a** Mild drought stress (T1) vs. normal water supply (CK); **b** moderate drought stress (T2) vs. CK; and **c** severe drought stress (T3) vs. CK. X-Axis indicates miRNA enrichment factors; y-axis corresponds to miRNA-enriched metabolic pathways

The results of KEGG analysis of miRNAs are shown in Additional file 10: Table S7. The top 20 enriched pathways are shown in Additional file 11: Table S8 and some KEGG scatter diagrams and pathways are shown in Fig. 7 and Additional file 12: Figure S4. The top five pathways based on KEGG enrichment factors were as follows: D-alanine metabolism, sulfur relay system, sulfur metabolism, ascorbate and aldarate metabolism, and mineral absorption for CK vs. T1; D-alanine metabolism, sulfur metabolism, mineral absorption, ascorbate and aldarate metabolism, and alpha-linolenic acid metabolism for CK vs. T2; D-alanine metabolism, mineral absorption, photo transduction, sulfur relay system, and porphyrin and chlorophyll metabolism for CK vs. T3; D-alanine metabolism, lipoic acid metabolism, mineral absorption, sulfur metabolism, and porphyrin and chlorophyll metabolism for T1 vs. T2; D-alanine metabolism, mineral absorption, photo transduction, porphyrin and chlorophyll metabolism, and Ca^2+^ signaling pathway for T2 vs. T3; and D-alanine metabolism, mineral absorption, photo transduction, porphyrin and chlorophyll metabolism, and sulfur relay system for T1 vs. T3. The most heavily enriched pathway in all groups was D-alanine metabolism, suggesting that this process plays an essential role in the drought stress response. The differential expression of sulfur metabolism (including the sulfur relay system) and mineral absorption pathways in all groups implied these pathways also play key roles in the drought stress response. Metabolic pathways related to D-alanine metabolism, sulfur metabolism, and mineral absorption were significantly affected when tea plants were subjected to mild and moderate drought stress (T1 and T2) compared with CK. Metabolic pathways related to photosynthesis were significantly affected under severe drought stress (T3), suggesting that only severe drought stress significantly affects photosynthesis in tea plants. Liang [44] found that drought stress increased D-alanine synthase activity in tobacco. Sulfur was found to be a significant determinant of drought stress tolerance in *Brassica napus* [45]. Some minerals have been shown to enhance the resistance of plants to drought stress [46]. On the basis of our results, we inferred that applications of D-alanine, sulfur, and minerals may help to alleviate drought stress in tea plants.

**Fig. 8:**
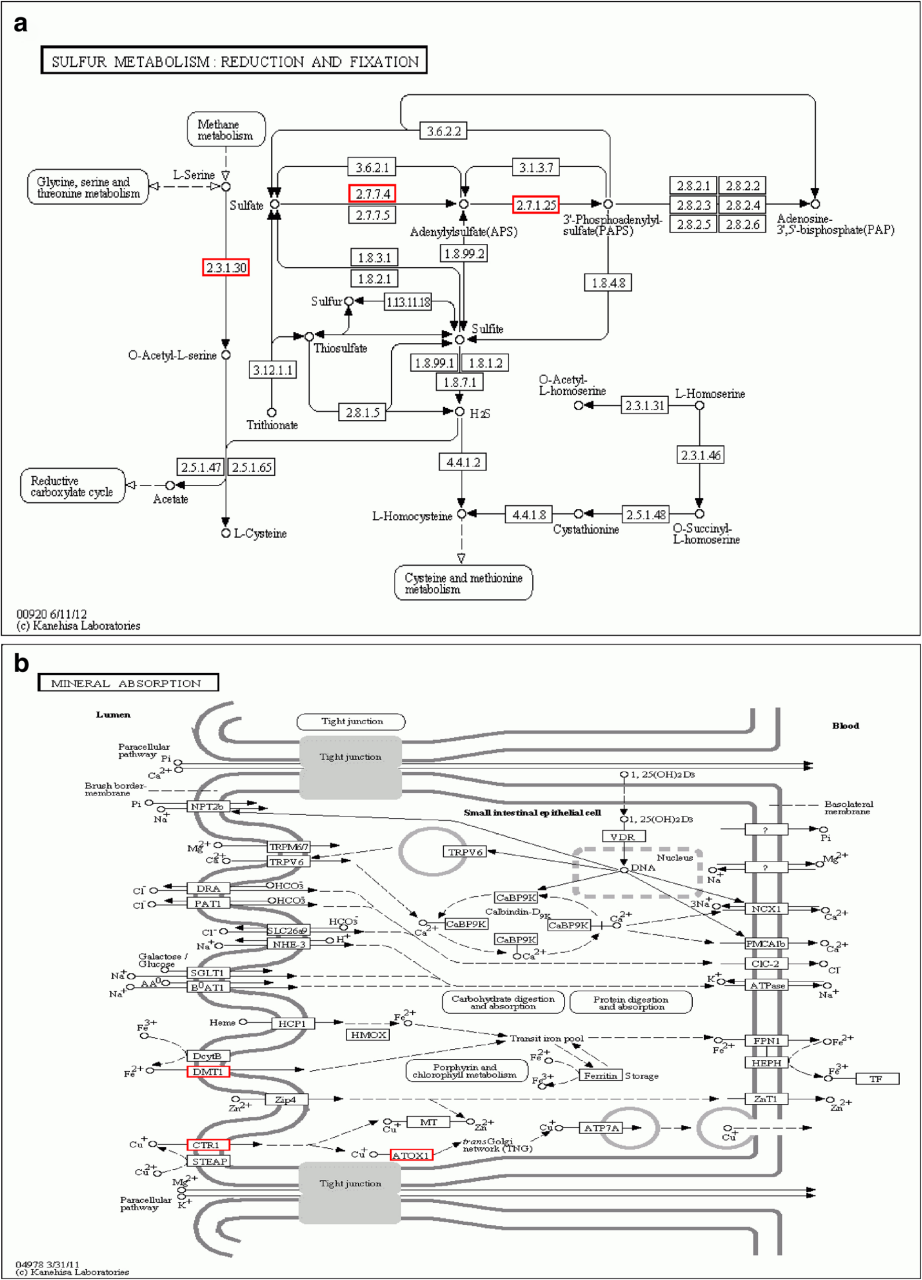
Targets of differentially expressed miRNAs in enriched pathways of sulfur metabolism and mineral absorption. **a** Schematic of sulfur metabolism pathway showing targets of miRNAs differentially expressed between normal water supply (CK) and mild (T1), moderate (T2), and severe (T3) drought conditions. **b** Schematic of mineral absorption pathway showing targets of miRNAs differentially expressed between normal water supply and mild, moderate, and severe drought conditions. Numbers in boxes are Enzyme Commission (EC) numbers. No map of D-alanine metabolism pathway and associated targets is shown

Sulfur metabolism and mineral absorption, the key enriched pathways along with D-alanine metabolism in CK vs. T1, CK vs. T2, and CK vs. T3, are illustrated in Fig. 8. Only one target gene of differentially expressed miRNAs was predicted: a gene encoding the 50S ribosomal protein (GenBank accession number HP752910.1) targeted by miRNAs such as miR854, miR1858a, and miR530-5p. A previous study showed that the 50S ribosomal protein enhanced the drought resistance of barley by accelerating degradation of abnormal proteins under drought conditions [47]. In this study, we identified three genes related to the sulfur metabolism pathway that were targeted by drought-related miRNAs: serine acetyltransferase (EC 2.3.1.30) gene targeted by miR5563-5p and miR159a; ATP sulfurylase (EC 2.7.7.4) gene with seven members targeted by miR395a; and APS kinase (EC 2.7.1.25) gene with seven members targeted by miR395a. Sulfur has been shown to be a pivotal component in plant responses to abiotic stresses [48], suggesting that these targets of differentially expressed miRNAs play key roles in sulfur assimilation during drought stress in tea plants.

The five genes targeted by miR854 were associated with the mineral absorption pathway: one gene (*DMT1*) encoding a divalent metal-ion transporter-1 (AT1G47240.1), three *CTR* genes encoding copper transporters (KA299366.1, KA292949.1 and HP749962.1), and the *ATOX1* gene (KA286605.1). *DMT1* is highly relevant to mineral homeostasis [49] and *CTR1* functions in maintaining copper homeostasis in different plant species [50]. *ATOX1* is a copper metallochaperone protein. Therefore, copper homeostasis might be very important during the drought stress response in tea plants.

### Quantitative expression analysis of miRNAs during drought stress in *C. sinensis*

**Fig. 9:**
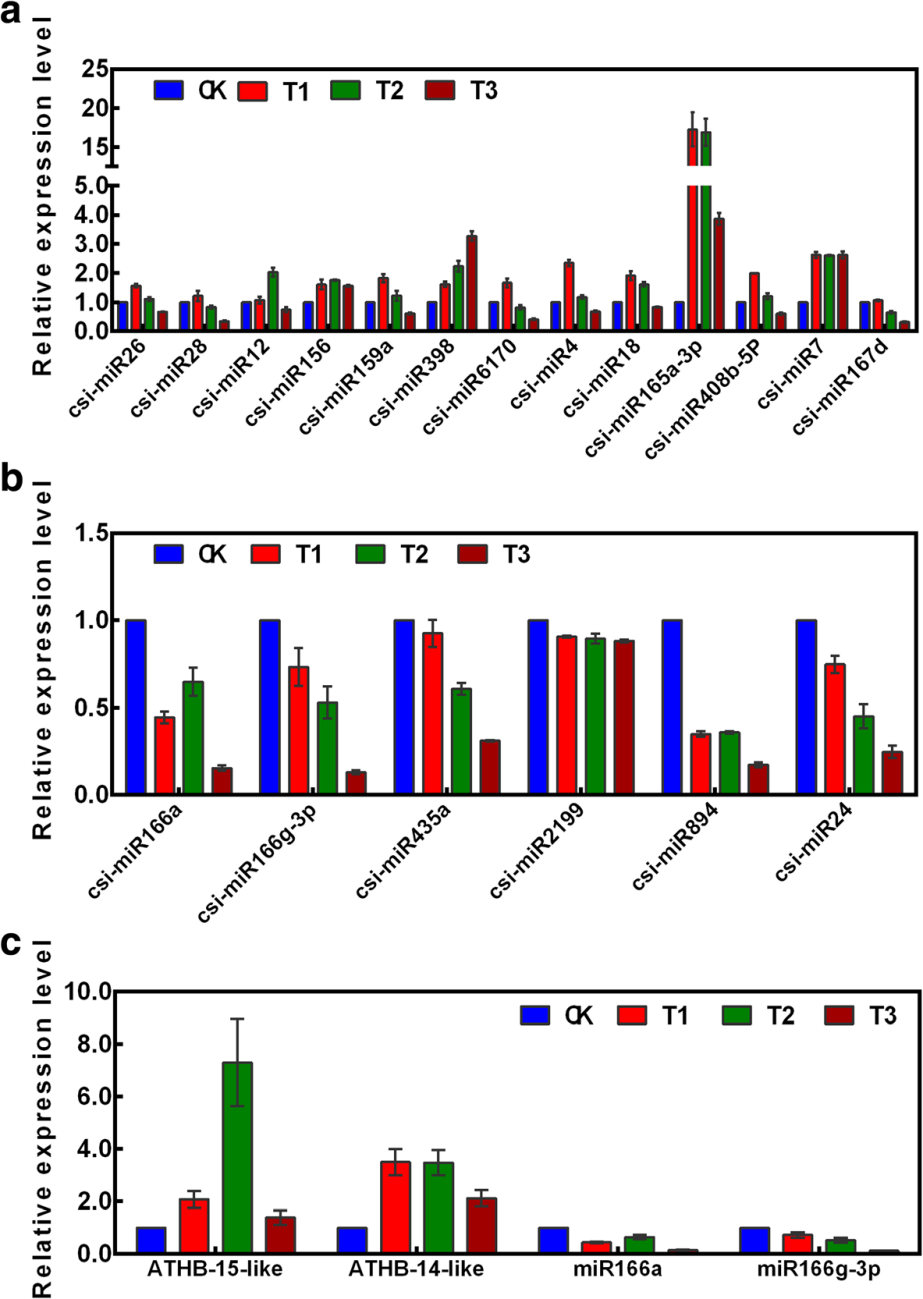
Expression profiles of selected miRNAs and targets under different drought stress conditions. **a**–**b** Expression profiles of 21 selected conserved (A) and novel (B) miRNAs under normal water supply (CK), mild drought (T1), moderate drought (T2), and severe drought (T3) conditions. **c** Expression profiles of miR166s and their target genes (*ATHB-14-like* and *ATHB-15-like*) under CK, T1, T2, and T3 conditions. Microarray expression signals were obtained with three probe repeats. Error bars indicate SE based on three replicates

The high-throughput sequencing results revealed differential expression of miRNAs under drought stress, and further analyses predicted the functions of the target genes. On the basis of those results, we selected 14 conserved miRNAs (csi-miR156, csi-miR159a, csi-miR165a-3p, csi-miR854, csi-miR166a, csi-miR166g-3p, csi-miR167d, csi-miR2199, csi-miR398, csi-miR408b-5p, csi-miR435a, csi-miR6170, csi-miR894, and csi-miR395a) and seven novel miRNAs (csi-miR4, csi-miR7, csi-miR12, csi-miR18, csi-miR24, csi-miR26, and csi-miR28), all possibly associated with the *C. sinensis* drought stress response, for validation of their expression profiles. In the qPCR analysis, expressions of all selected miRNAs were detected in the various treatments. According to their expression in T1 compared with that in CK, the 21 miRNAs could be divided into two groups: group 1, which showed higher expression in T1 than in CK (Fig. 9a), and group 2, which showed lower expression in T1 than in CK (Fig. 9b).

Group 1 consisted of 13 miRNAs: csi-miR26, csi-miR28, csi-miR12, csi-miR156, csi-miR159a, csi-miR398, csi-miR170, csi-miR4, csi-miR18, csi-miR165a-3p, csi-miR408b-5p, csi-miR7, and csi-miR854 (Fig. 9a). Two miRNAs, namely csi-miR398 (conserved miRNA) and csi-miR7 (novel miRNA), were up-regulated during drought stress. The expression level of csi-miR398, which increased linearly in all samples, differed significantly among treatments. The expression level of csi-miR7 was significantly higher in T1 than in CK, but not significantly different among T1, T2, and T3. The six conserved miRNAs (csi-miR156, csi-miR159a, csi-miR6170, csi-miR165a-3p, csi-miR408b-5p, and csi-miR854) and five novel miRNAs (csi-miR26, csi-miR28, csi-miR12, csi-miR4, and csi-miR18) were up-regulated and then down-regulated in *C. sinensis* under drought stress. The expression levels of csi-miR165a-3p, csi-miR159a, csi-miR408b-5p, csi-miR6170, and csi-miR854 were the highest in T1 and lowest in T3. The expression level of miR156 increased under drought stress, similar to the expression pattern of miR156 in *A. thaliana* under high salt and low temperature stresses [51]. The novel miRNAs csi-miR4, csi-miR18, csi-miR26, and csi-miR28 showed the highest expression levels in T1 and the lowest expression levels in T3.

Group 2 contained eight miRNAs: the conserved miRNAs csi-miR166a, csi-miR166g-3p, csi-miR435a, csi-miR2199, csi-miR894, csi-miR167d, and csi-miR395a; and the novel miRNA csi-miR24. These miRNAs were down-regulated in *C. sinensis* during drought stress (Fig. 9b), implying that they were under negative regulation. The lowest expression levels of these miRNAs were in T3. Among these miRNAs, miR166 is a highly conserved drought-responsive miRNA [52–57] that is also known to be involved in root tip development. This implies that post-transcriptional regulation mediated by miR166 is a crucial regulatory pathway involved in plant root architecture and the drought response.

### Phase-specific miRNAs during drought stress in *C. sinensis*

**Fig. 10:**
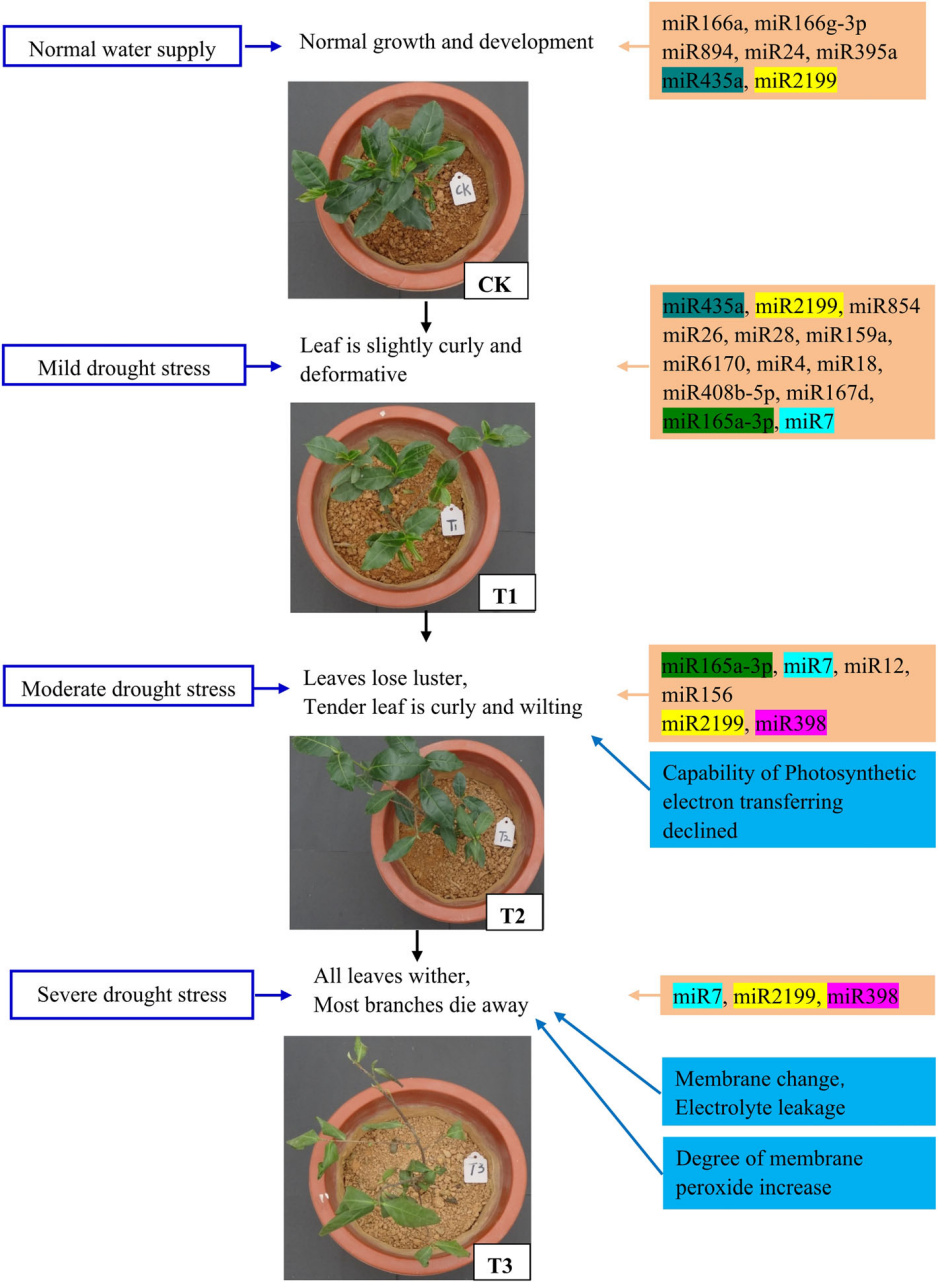
Functional mechanisms underlying physiological changes and phase-specific miRNAs during drought stress in *Camellia sinensis*

Based on our results, we constructed a diagram showing phase-specific miRNAs expressed under drought stress in *C. sinensis* (Fig. 10). Under normal water supply (CK), *C. sinensis* plants showed normal growth and development, and soil moisture content, leaf water content, electrical conductivity, and chlorophyll and MDA concentrations were all at normal levels. Seven miRNAs (miR166a, miR166g-3p, miR435a, miR894, miR2199, miR24, and miR395a) showed significantly higher expression levels in CK than in the T1, T2, and T3 treatments.

Under mild drought stress (T1), the leaves of *C. sinensis* plants showed slight curling and deformation; soil moisture, leaf water content, and chlorophyll (chlorophyll a, chlorophyll b and total chlorophyll) concentrations decreased, while leaf electrical conductivity and MDA concentration were barely affected. The 11 miRNAs (miR26, miR28, miR159a, miR6170, miR4, miR18, miR165a-3p, miR408b-5p, miR7, miR854, and miR167d) showed maximum expression levels in T1. The expression levels of miR435a and miR2199 were also relatively high in T1, compared with CK.

In T2, there was a further decrease in soil moisture content and the *C. sinensis* plants showed a loss of leaf luster, curling and wilting of tender leaves, decreased leaf water content and chlorophyll concentration, and slight increases in leaf electrical conductivity and MDA concentration. These changes reflected a declining photosynthetic electron transfer capability. The two miRNAs, miR12 and miR156, showed maximum expression levels in T2, and the expression levels of miR165a-3p, miR7, miR2199, and miR398 were also high.

In T3, leaves of *C. sinensis* plants withered completely and most branches died. Soil moisture, leaf water content, and leaf chlorophyll concentration continued to decline. In addition, the leaf MDA concentration and electrical conductivity markedly increased. The significant increase in cell electrolyte leakage was indicative of disruptions to plant cell structure and functional integrity, which ultimately affect plant growth and development. In T3, the only miRNA showing maximum expression was miR398, although there were also high expression levels of csi-miR7 and miR2199. These results were consistent with those of the KEGG analysis, that metabolic pathways related to photosynthesis were significantly affected under T3. Since photosynthesis was not affected in the T1 and T2 treatments, these results suggested that photosynthesis in *C. sinensis* may not be significantly affected below a certain threshold of drought stress.

### Cloning and validation of miR166 target genes in *C. sinensis*

To explore the regulatory functions of csi-miR166, we cloned the HD-Zip III subfamily transcription factors *ATHB-14-like* and *ATHB-15-like*, and then monitored their expression patterns under drought stress by qPCR. Both of these transcription factors were up-regulated under T1, T2, and T3 (Fig. 9c). Interestingly, the highest expression level of *ATHB-15-like* was in T2, whereas *ATHB-14-like* was induced and maintained at higher levels under T1 and T2 than under T3. In contrast, miR166a and miR166g-3p were down-regulated under T1, T2, and T3, indicating that the expression levels of miR166s were negatively correlated with those of their target genes.

## Discussion

### Variation in sRNA length distribution and number of miRNAs in *C. sinensis* during drought stress

Analyses of *C. sinensis* sRNA length distributions in the absence of reference genome sequence calibrations revealed that the most common sRNA length in the four libraries was 24 nt, followed by 21 nt. These sRNA lengths, which varied during drought stress, are similar to those previously reported for plants such as *B. napus* [58] The 24-nt sRNAs accounted for 71.48%, 65.87%, 64.63%, and 42.26% of total sRNAs in CK, T1, T2, and T3 samples, respectively. The proportion of 21-nt sRNAs was 11.50% (CK), 15.02% (T1), 15.57% (T2), and 33.25% (T3), indicating that an increasing number of 21-nt sRNAs participate in biological regulation during drought stress in *C. sinensis* plants. Meanwhile, the proportion of miRNAs and the number and percentage of unique miRNAs tended to increase under drought stress (see data in Table 2). Thus, we inferred that Tieguanyin tea plants respond to drought stress by synthesizing and activating more miRNAs involved in drought regulation.

By using the genome of the model plant *A. thaliana* as a reference and comparing our sequence data with genetic data in the GenBank and Rfam databases, we annotated 883,059 (5.60%), 970,915 (7.35%), 1,110,918 (7.66%), and 2,122,227 (15.78%) miRNAs in the CK, T1, T2, and T3 libraries, respectively. Further comparison of miRNA sequencing data from the drought-treatment libraries with information in the *C. sinensis* transcriptome database led to the discovery of additional novel miRNAs and the precise locations of their target loci in the Tieguanyin tea plant genome. In total, 176 miRNA sequencing reads were identified and 4067 target loci were predicted.

Although many novel miRNAs were annotated in the four drought-stress libraries, some miRNAs remained unidentified, as has been the case in studies on other plants [59–61].

### Drought-related miRNAs in *C. sinensis*

To elucidate the drought-resistance mechanisms *C. sinensis,* it is important to explore the spatio-temporal variations in miRNAs under drought stress. In this study, most miRNAs showed low abundance in drought-stressed *C. sinensis,* and only a few miRNA families were over-expressed, consistent with the results of other studies on plant miRNAs [62, 63]. We also observed considerable differences in expression levels of miRNAs in the same family, such as miR166a and miR166g-3p. The expression patterns of miRNAs are highly conserved among different plants. For example, members of the miR156, miR166, and miR398 families are over-expressed in most plants [64–66]. Our results indicated that the expression patterns of these miRNAs in *C. sinensis* are similar to those in other plants. The results of this study revealed a relationship between stress resistance and conserved miRNAs such as miR156, miR166a, and miR398. This relationship has also been observed in other plants [27, 67, 68], suggesting that these miRNAs might play key roles in the drought stress response in *C. sinensis*. The correlation between miRNAs and the drought stress response of *C. sinensis* requires further verification.

### Functions of genes targeted by drought-related miRNAs in *C. sinensis*

Among the miRNAs identified in our study, miR398 plays a crucial regulatory role in respiration by targeting cytochrome c oxidase subunit V, an enzyme involved in electron transport in the mitochondrial respiratory pathway [69, 70]. Various studies have shown that miR398 is up-regulated under drought stress in *Triticum dicoccoides* [19], down-regulated in *Zea mays* [14], and both up-regulated and down-regulated in *Medicago truncatula* [16, 20]. In our study, miR398 was up-regulated in *C. sinensis* during drought stress, consistent with miR398 expression patterns observed in *M. truncatula* [16] and *T. dicoccoides* [19]. In a previous study, decreased miR398 expression levels coupled with increasing levels of Cu/Zn-superoxide dismutases (Cu/Zn-SOD) in wild *Ipomoea campanulata* under drought stress confirmed the role of miR398 in drought stress tolerance. In another study, however, expression of miR398 did not correlate with Cu/Zn-SOD accumulation in cultivated *Jacquemontia pentantha* [71]*.* These contrasting results indicate that the functions of miR398 differ among species, with the metabolic status of individual plants, and with the severity and duration of drought stress. Therefore, the regulatory mechanisms of miR398 in plants are still unknown.

One of the most highly conserved miRNAs, miR156, regulates the timing of developmental transitions [72]. Members of the miR156 family are known to be highly induced by heat stress and to play roles in heat stress memory in plants such as wheat (*T. aestivum*) [73], *B. rapa* [74], and *A. thaliana* [75]. Previous studies have found that csi-miR156 regulates *DFR* transcription by controlling *SPL* expression to influence catechin biosynthesis [76–78]. The accumulation of plant secondary metabolites is closely related to stress resistance in plants. We observed that miR156 was up-regulated under drought stress in *C. sinensis*, suggesting that this miRNA activates the catechin biosynthesis pathway in tea plants during drought stress. We inferred that miR156 enhances the resistance of tea plants to drought stress by inducing the accumulation of secondary metabolites.

Reyes et al. [79] reported that miR159 expression increased in germinating seeds of *Arabidopsis* treated with ABA and drought. Further studies showed that miR159a mediates the cleavage of *MYB33* and *MYB101* transcripts in *A. thaliana* [79, 80], while *MYB* transcription factors bind *cis-*elements in the promoter of the dehydration-responsive gene dehydration 22 (*RD22*) and activate *RD22* cooperatively. Osmotic stress tolerance in transgenic plants can be improved by over-expression of both *MYC2* and *MYB2* [81]. Microarray analyses have shown that miR167 is induced and up-regulated during drought stress in *A. thaliana* [51]. As a positive regulator of drought stress resistance, phospholipase D (*PLD*) is a predicted target of miR167. In *Z. mays*, the accumulation of *PLD* mRNA was shown to increase as the expression of miR167 decreased under drought stress [14]. Zhou et al. [18] reported that miR854 played a regulatory role in the drought stress response of rice, but its mechanism was unclear. In our study, the expression levels of miR167 and miR854 in *C. sinensis* increased under mild drought stress, but decreased with increasing severity of drought stress (Fig. 9a and b). Thus, we hypothesize that mild drought stress up-regulates miR167 and miR854.

In our study, csi-miR166a, csi-miR166g-3p, csi-miR435a, csi-miR894, csi-miR2199, csi-miR24, and csi-miR395a were down-regulated in *C. sinensis* during drought stress. The expression pattern of miR166 in response to drought stress differs among plant species; it is down-regulated in barley [15] and *T. dicoccoides* [19] but up-regulated in *M. truncatula*, especially in roots [16]. According to other reports, miR166a over-expression reduces the number of lateral roots in *M. truncatula* [82] and strengthens vascular development and enlarge shoot apical meristems in *A. thaliana* [83]. A previous study indicated that limitation of external water supply changes the architecture of the plant root system to improve water absorption efficiency [84]. In the present study, the miR166 family members csi-miR166a and csi-miR166g-3p were found to negatively regulate their target genes in *C. sinensis* during drought stress. This pattern of regulation is consistent with those observed in cotton under salt stress [63] and in wheat under drought stress shock [19]. The co-adjustment of the miR166 family and its targets *ATHB-14-like* and *ATHB-15-like* indicate the existence of negative feedback regulation in the miRNA pathway. We observed the strongest up-regulation of *ATHB-15-like* under moderate drought stress, indicating that the miR166 family strongly regulated its expression at this stage. This result implies that the expression of target genes of miR166 family members is regulated by dynamic changes in expression levels of their corresponding miRNAs. To adapt to environmental changes, tea plants can correspondingly self-regulate in response to different levels of drought stress. The predicted target of miR395, a miRNA enriched under drought treatment, is the sulfate transporter *SULTR2* [85]. In regard to sulfate transporters, miR395 plays an important role in reequilibrating the sulfate flux in different tissues and improving drought stress resistance in plants [86, 87]. ATP sulfurylase has a crucial function in the formation of APS from ATP and sulfate [88, 89], a process catalyzed by APS kinase (EC 2.7.1.25; 7 unigenes) [90]. In our study, ATP sulfurylase and *APK* were up-regulated in tea plants under drought stress, and their encoding genes were predicted targets of miR395a.

### D-alanine metabolism, sulfur metabolism, and mineral absorption metabolism play important roles in the drought stress response of *C. sinensis*

Analyses of the target genes of miRNAs related to drought stress indicated that D-alanine metabolism, sulfur metabolism, and mineral absorption metabolism were highly enriched pathways. Thus, these pathways might play important roles in the drought stress response of *C. sinensis.* In some species, these pathways are involved the responses to various abiotic stresses. For example, the formation of free amino acids in cotton under high temperature stress may play a significant role in maintaining cell water potential, eliminating toxicity, and storing nitrogen [91]; sulfur metabolism was found to be an important component of the cold resistance mechanism of cassava [92]; in *B. napus* [45], sulfur uptake affected the availability of total sulfur, which played important roles in alleviating damage caused by drought stress. Some minerals have been shown to enhance the drought tolerance of plants by increasing the concentration of antioxidants [46]. Therefore, D-alanine metabolism, sulfur metabolism, and mineral absorption pathways are likely to play important roles in the drought stress response of *C. sinensis*. Interestingly, a role of D-alanine metabolism in plant abiotic stress responses has seldom been reported, and warrants further research.

Under drought stress, a series of physiological, biochemical, and molecular changes lead to adaptive drought responses in plants [93, 94]. Previous studies have found that drought stress affects carbon dioxide assimilation rates and photosynthetic pigment synthesis [93, 95] and results in the generation of reactive oxygen species that cause oxidative damage as measured by lipid peroxidation [96]. We observed that the expression of miRNAs in *C. sinensis* differed significantly among drought treatments with different degrees of severity. Some specific metabolic pathways activated under drought stress in tea plants were identified. These findings provide new clues about the molecular mechanism of the drought stress response in tea plants.

## Conclusions

We analyzed drought-responsive miRNAs in tea plants, and found that most of their targets were related to transcriptional regulation. The most highly enriched pathways under drought stress were D-alanine metabolism, sulfur metabolism, and mineral absorption metabolism. Based on the results of qPCR analyses, 21 miRNAs associated with the drought stress response were divided into two groups. The results of this study showed that the expressions of phase-specific miRNAs vary with morphological, physiological and biochemical changes that occur under drought stress. These findings will be useful for research on drought resistance and provide insights into the mechanisms of drought adaptation and resistance in *C. sinensis*.

## Additional files

Additional file 1: Table S1. Primers used for qPCR amplification of *Camellia sinensis* ‘Tieguanyin’ miRNAs. (DOCX 14 kb)

Additional file 2: Table S2. Summary of novel miRNA targets predicted from Tieguanyin RNA sequences generated under different drought stress conditions. (DOCX 13 kb)

Additional file 3: Figure S1. Heat map of *Camellia sinensis* novel miRNAs differentially expressed between different drought stress conditions (CK, normal water supply; T1, mild drought stress; T2, moderate drought stress; T3, severe drought stress). (JPEG 1290 kb)

Additional file 4: Table S3. *Camellia sinensis* known miRNAs differentially expressed between normal water supply and mild drought stress (CK vs. T1). (XLSX 14 kb)

Additional file 5: Table S4. *Camellia sinensis* known miRNAs differentially expressed between normal water supply and moderate drought stress (CK vs. T2). (XLSX 15 kb)

Additional file 6: Table S5. *Camellia sinensis* known miRNAs differentially expressed between normal water supply and severe drought stress (CK vs. T3). (XLSX 21 kb)

Additional file 7: Figure S2. Gene Ontology annotations of predicted target genes of miRNAs identified from mild drought stress treatment (T1). (JPEG 2589 kb)

Additional file 8: Figure S3. Gene Ontology annotations of predicted target genes of miRNAs identified from moderate drought stress treatment (T2). (JPEG 2596 kb)

Additional file 9: Table S6. GO statistics of *Camellia sinensis* known miRNAs during drought stress. (XLS 32 kb)

Additional file 10: Table S7. KEGG pathways enriched in *Camellia sinensis* known miRNAs during drought stress. (XLS 984 kb)

Additional file 11: Table S8. Top 20 KEGG pathways enriched in *Camellia sinensis* known miRNAs during drought stress. (XLS 167 kb)

Additional file 12: Figure S4. Statistical summary of KEGG enriched pathways of top 20 target genes of novel miRNAs. (JPEG 5124 kb)

## Abbreviations

ABA: Abscisic acid
cDNA: Complementary DNA
DNA: Deoxyribonuclease acid
h: Hour
MDA: Malondialdehyde
MFE: Minimum free energy
miRNA: microRNA
mRNA: messenger RNA
PCR: Polymerase chain reaction
qPCR: Real time quantitative PCR
RNA: Ribonuclease acid
rRNA: Ribonuclease RNA
s: Second
scRNA: Small cytosol RNA
snoRNA: Small nucleolar RNA
snRNA: Small nuclear RNA
tRNA: Transfer RNA
