# Supplementary material for: Identification of drought-responsive miRNAs and physiological characterization of tea plant (Camellia sinensis L.) under drought stress
Source: BMC Plant Biol. 2017 Nov 21;17:211. doi: 10.1186/s12870-017-1172-6 (PMC5696764; doi:10.1186/s12870-017-1172-6)
Supplement: Supplementary file 1 — Primers used for qPCR amplification of Camellia sinensis ‘Tieguanyin’ miRNAs. (DOCX 14 kb) [file 12870_2017_1172_MOESM1_ESM.docx]

**Additional file 1: Table S1. Primers used for qPCR amplification of *Camellia sinensis* ‘Tieguanyin’ miRNAs.**

| miRNA | Prime sequences (5'-3') | Prime temperature | Amplification efficiency |
| --- | --- | --- | --- |
| csi-miR156 | CCTGACAGAAGAGAGTGAGCAC | 60 | 1.918 |
| csi-miR159a | TTTGGATTGAAGGGAGCTCTAG | 60 | 1.962 |
| csi-miR165a-3p | TCGGACCAGGCTTCATC | 58 | 1.999 |
| csi-miR166a | ACCAGGCTTCATTCCCC | 57 | 2.007 |
| csi-miR166g-3p | GGACCAGGCTTCATTCCTC | 60 | 1.990 |
| csi-miR167d | GAAGCTGCCAGCATGATCTAG | 57 | 1.933 |
| csi-miR2199 | GCCTGATAACTCGACGGATC | 60 | 2.004 |
| csi-miR398 | GGAGCGACTTGAGATCACATC | 60 | 2.098 |
| csi-miR408b-5p | ACGGGGATGAGACAGAGC | 59 | 2.057 |
| csi-miR435a | TGACAACGAGAGAGAGCACGC | 60 | 2.030 |
| csi-miR6170 | GGGCAGAAAAGTAGAGAGGTGA | 60 | 2.046 |
| csi-miR894 | GTTTCACGTCGGGTTCAC | 57 | 1.928 |
| csi-miR4 | GTGGTGGTGGTGGTGGTA | 58 | 2.073 |
| csi-miR7 | GCGTACGGGTTCATAGATG | 57 | 2.055 |
| csi-miR12 | TGTCGCGGATAATTTTGGC | 62 | 1.926 |
| csi-miR18 | TGGCCAAGTCAGGTTCTG | 58 | 1.974 |
| csi-miR24 | GGAATGTTGTCTGGCTCGAG | 61 | 2.060 |
| csi-miR26 | TTTTCCAAGACCACCCATGCC | 62 | 2.055 |
| csi-miR28 | GGTAGAGAGATGGTGGGAC | 60 | 2.053 |
| U6 | CGATACAGAGAAGATTAGCATGG | 59 | 1.923 |
| 5.8s rRNA | ACGTCTGCCTGGGTGTCACAA | 62 | 2.021 |
