# Supplementary material for: Identification of drought-responsive miRNAs and physiological characterization of tea plant (Camellia sinensis L.) under drought stress
Source: BMC Plant Biol. 2017 Nov 21;17:211. doi: 10.1186/s12870-017-1172-6 (PMC5696764; doi:10.1186/s12870-017-1172-6)
Supplement: Supplementary file 2 — Summary of novel miRNA targets predicted from Tieguanyin RNA sequences generated under different drought stress conditions. (DOCX 13 kb) [file 12870_2017_1172_MOESM2_ESM.docx]

**Additional file 2: Table S2. Summary of novel miRNA targets predicted from Tieguanyin RNA sequences generated under different drought stress conditions. The former data is the number of total novel miRNAs of Tieguanyin tea plant at different drought stress. The latter is the number of potential novel miRNAs that could be predicted target gene loci.**

| **Types** | **CK** | **T1** | **T2** | **T3** | **Total** |
| --- | --- | --- | --- | --- | --- |
| MiRNAs No. | 137/137 | 140/140 | 141/141 | 125/125 | 176/176 |
| Target gene loci No. | 3843 | 3907 | 3902 | 3296 | 4067 |
