# Supplementary figures and images for: Identification of drought-responsive miRNAs and physiological characterization of tea plant (Camellia sinensis L.) under drought stress
Source: BMC Plant Biol. 2017 Nov 21;17:211. doi: 10.1186/s12870-017-1172-6 (PMC5696764; doi:10.1186/s12870-017-1172-6)

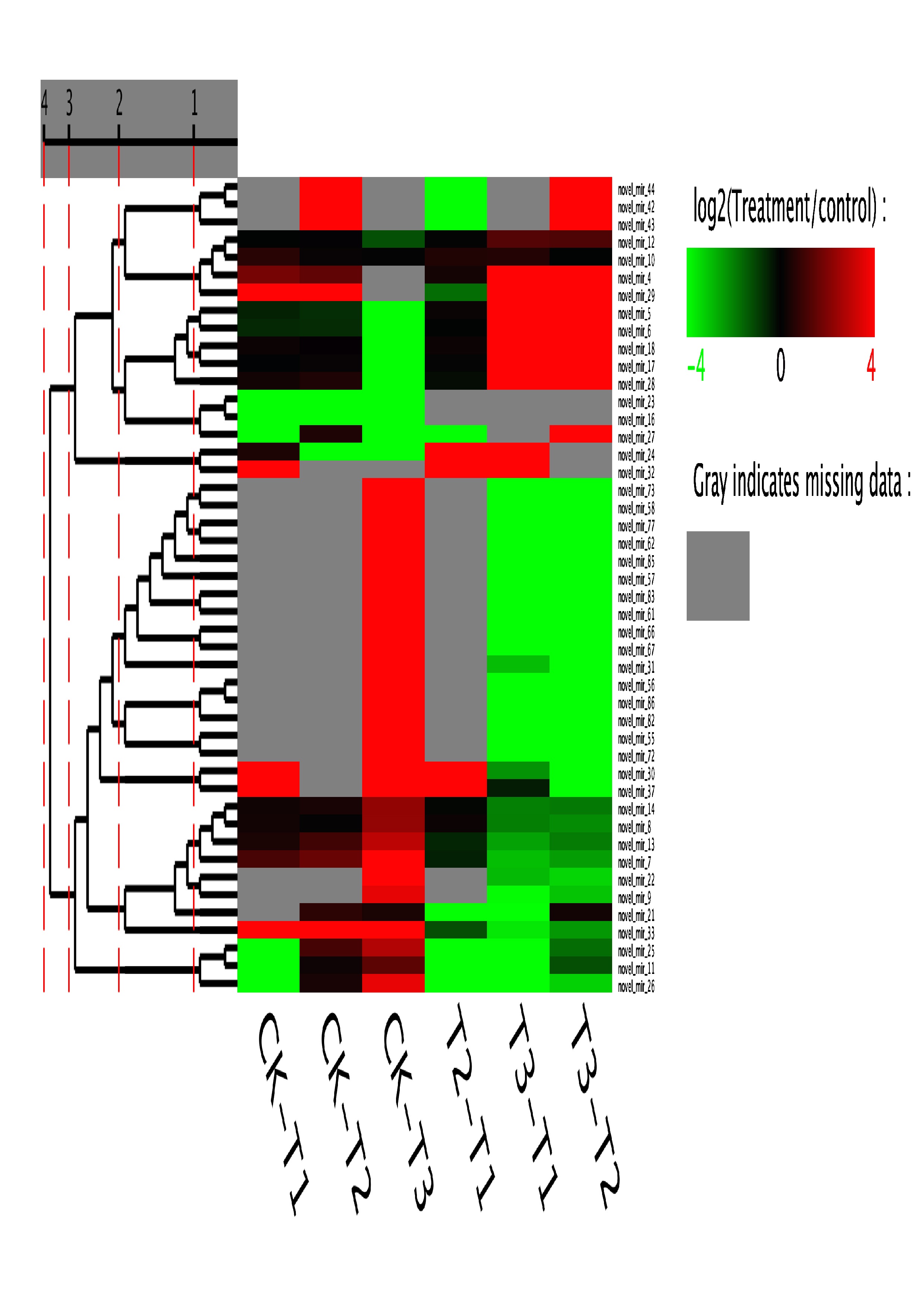

Supplement: Supplementary file 3 — Heat map of Camellia sinensis novel miRNAs differentially expressed between different drought stress conditions (CK, normal water supply; T1, mild drought stress; T2, moderate drought stress; T3, severe drought stress). (JPEG 1290 kb) [file 12870_2017_1172_MOESM3_ESM.jpg]

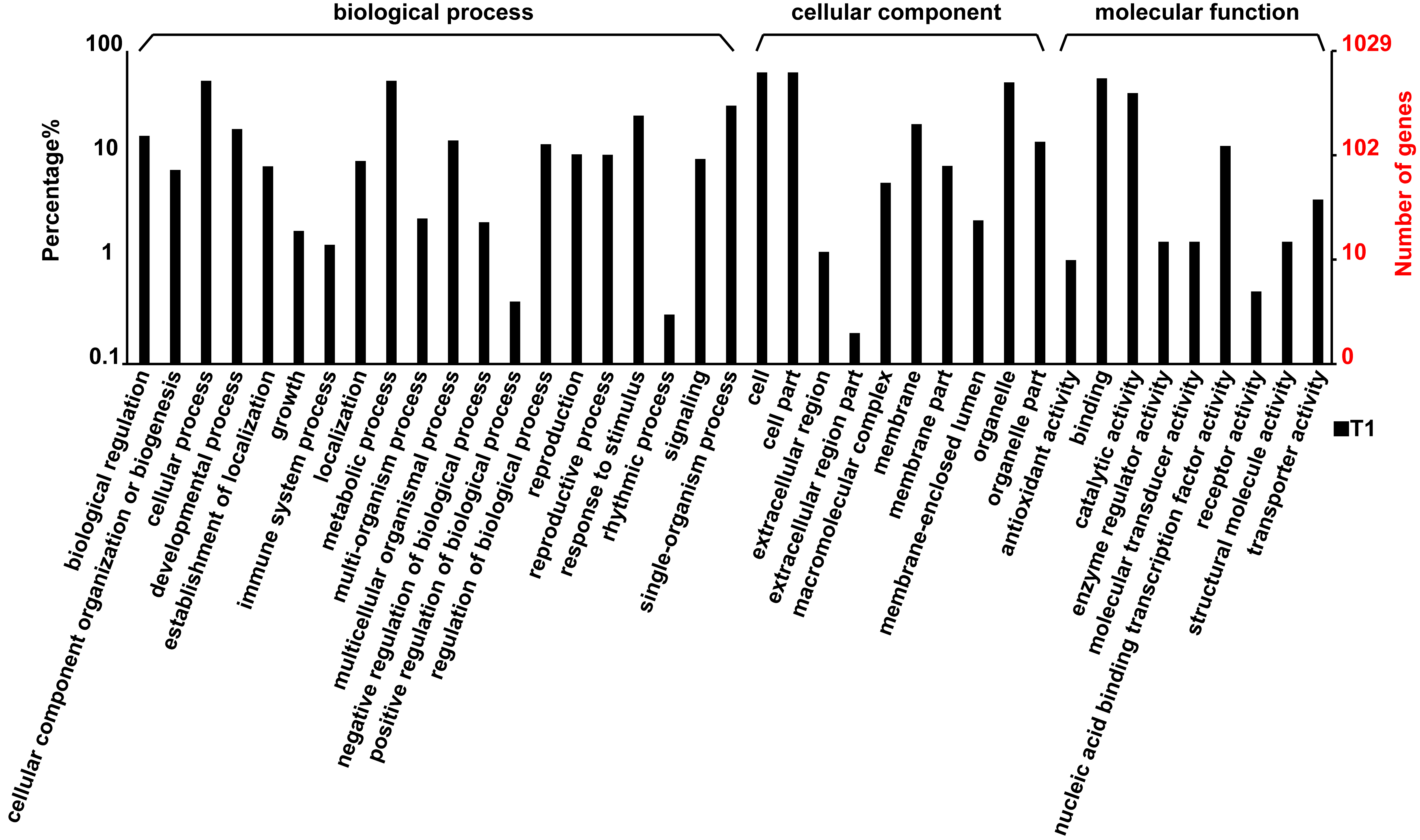

Supplement: Supplementary file 7 — Gene Ontology annotations of predicted target genes of miRNAs identified from mild drought stress treatment (T1). (JPEG 2589 kb) [file 12870_2017_1172_MOESM7_ESM.jpg]

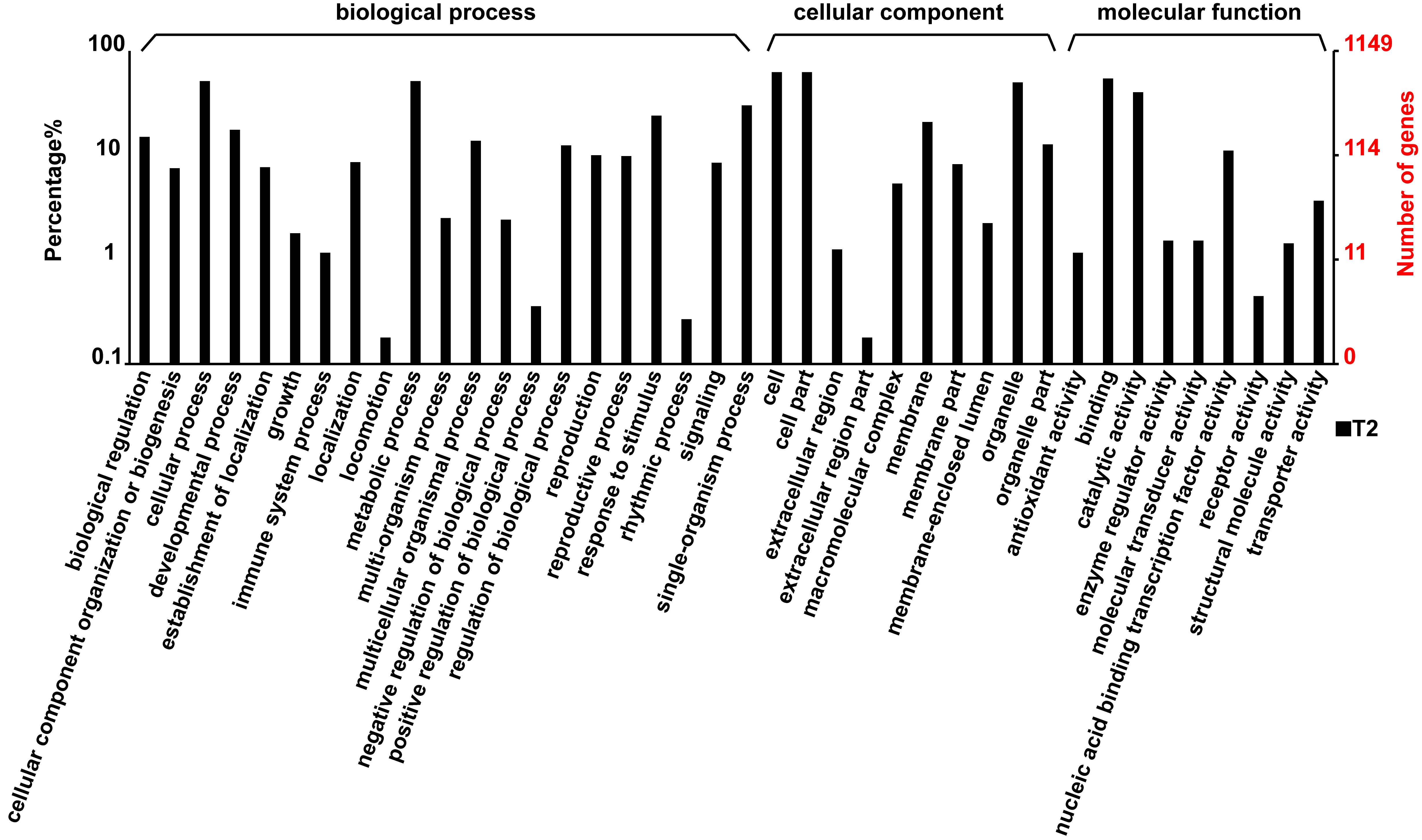

Supplement: Supplementary file 8 — Gene Ontology annotations of predicted target genes of miRNAs identified from moderate drought stress treatment (T2). (JPEG 2596 kb) [file 12870_2017_1172_MOESM8_ESM.jpg]

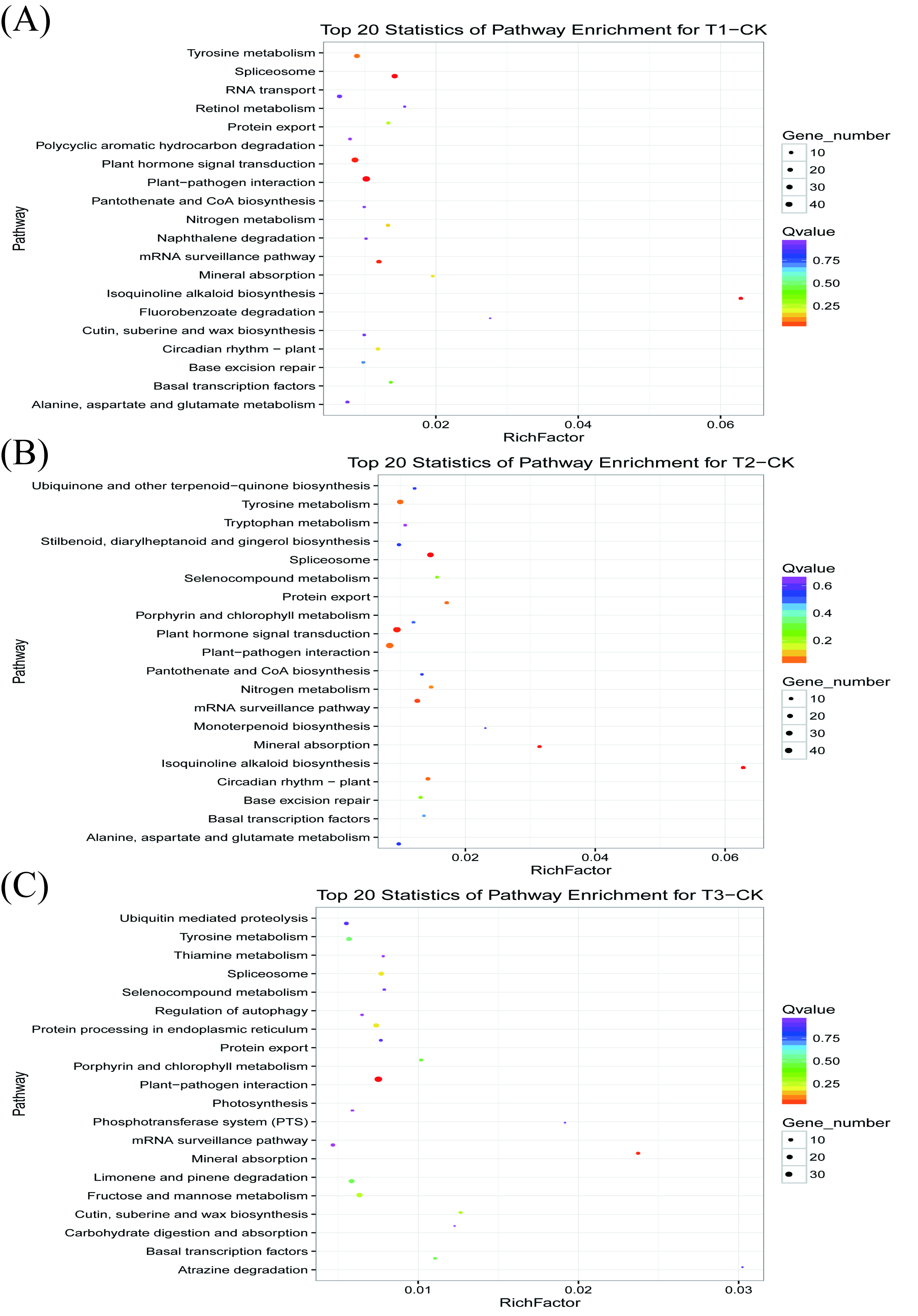

Supplement: Supplementary file 12 — Statistical summary of KEGG enriched pathways of top 20 target genes of novel miRNAs. (JPEG 5124 kb) [file 12870_2017_1172_MOESM12_ESM.jpg]
